# Evaluation and Treatment of Severe Rhabdomyolysis in a Patient with Legionnaires' Disease

**DOI:** 10.7759/cureus.5773

**Published:** 2019-09-26

**Authors:** Joshua W Buzzard, Zachary Zuzek, Ben P Alencherry, Clifford D Packer

**Affiliations:** 1 Miscellaneous, Case Western Reserve University School of Medicine, Cleveland, USA; 2 Internal Medicine, University Hospitals Cleveland Medical Center/Case Western Reserve University School of Medicine, Cleveland, USA; 3 Internal Medicine, Case Western Reserve University School of Medicine, Cleveland, USA

**Keywords:** legionella pneumophilia, legionella, rhabdomyolysis, legionnaires' disease, early diagnosis, intravenous fluid

## Abstract

A 53-year-old man with alcoholism and a three-day history of diarrhea and abdominal pain was hospitalized with mild acute kidney injury (AKI) and rhabdomyolysis after a fall where he was down for a short duration. Subsequent testing revealed patchy right lower lobe infiltrates on chest X-ray and a positive urinary Legionella antigen test. Creatinine phosphokinase (CPK) peaked at 85,780 U/L (normal 0-250) on hospital day two and remained markedly elevated for five days despite aggressive intravenous (IV) hydration and appropriate antibiotic treatment. When the patient defervesced and showed clinical signs of resolution of pneumonia, the CPK level declined rapidly, and renal function returned to baseline. Rhabdomyolysis with AKI is a rare but serious complication of Legionella pneumonia, with most patients requiring dialysis. Our patient’s complete recovery without renal replacement therapy can probably be attributed to his normal baseline renal function, timely diagnosis of his Legionella-associated rhabdomyolysis, and prompt treatment with aggressive IV hydration and appropriate antibiotics. Legionella infection should be considered in acutely ill patients with rhabdomyolysis of unclear etiology.

## Introduction

Legionella pneumophila infection can present in the context of an acute febrile illness (Pontiac fever), or pneumonia (Legionnaires' disease). Findings associated with Legionnaires' disease include gastrointestinal (GI) symptoms (nausea, diarrhea), hyponatremia, transaminitis, high fever, and neurological signs such as headache and confusion. Of the many problems associated with Legionnaires' disease, rhabdomyolysis accounts for a large portion of the morbidity and mortality in patients with this condition. Thus, it is important to recognize Legionella early as a potential causative agent in a patient presenting with acute illness and rhabdomyolysis. We report the case of a man with Legionnaires' disease who, with quick diagnosis and prompt administration of high volume intravenous (IV) fluids and appropriate antibiotics, avoided severe kidney injury despite significant elevation in creatinine phosphokinase (CPK).

## Case presentation

**Figure 1:**
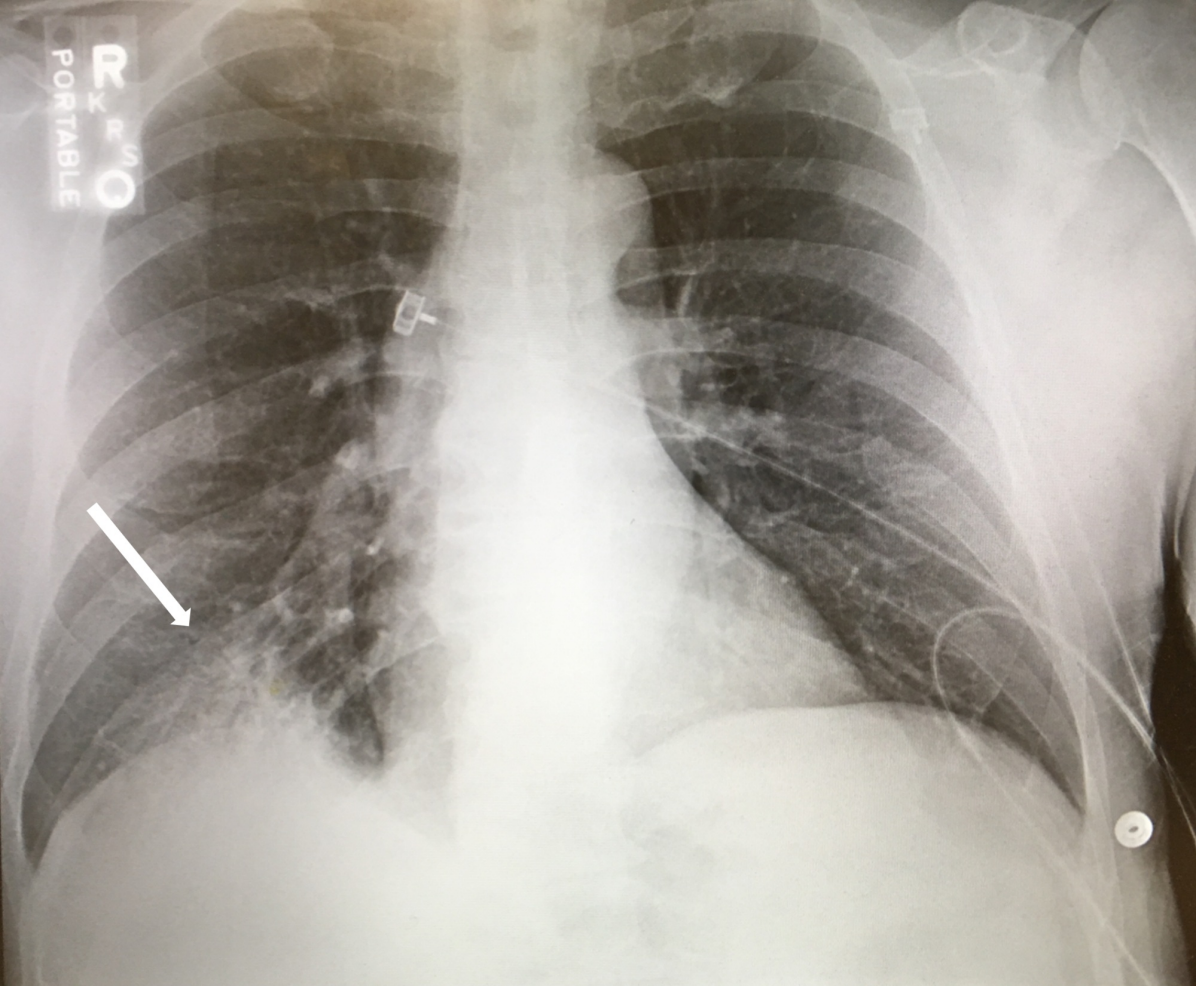
Chest X-Ray Chest X-Ray demonstrating patchy right lower lobe infiltrates.

A 53-year-old man lacking medical care for seven years presented to the emergency department following a fall at home. His past medical history was significant for alcohol use disorder, consuming eight to ten or more drinks per day; his last drink was the evening prior to admission. Previous attempts at alcohol cessation were unsuccessful due to strong withdrawal symptoms, but there was no history of seizures or delirium tremens. When he fell, he had hit his head on the wall, and endorsed being down for one hour with no loss of consciousness. He also complained of three days of copious, watery, nonbloody diarrhea, abdominal pain, nausea, and poor per os (PO) intake. He denied any subjective fevers, chills, night sweats, vomiting, headache, confusion, sick contacts, or recent change in diet. He was a nonsmoker. Initial vital signs were body temperature 99.3 °F, pulse rate 115 bpm, blood pressure 165/87 mmHg, and respiration rate 18. Physical exam was unremarkable aside from rales heard on auscultation and positive egophany of the right lower lung lobe. There was no accessory muscle use, no general muscle tenderness or ecchymoses noted. Initial lab tests were significant for ethyl alcohol (EtOH) level 87 mg/dL (normal 0-10) with urine drug screen positive only for ethanol, serum sodium 123 mmol/L (136-148), bicarbonate 18 mmol/L (21-32), blood urea nitrogen (BUN) 21 mg/dL (8-20), creatinine 1.3 mg/dL (0.5-1.2), and troponin 0.1 ng/dL (0-0.05). The patient's baseline levels were largely unknown due to his infrequent health care use. Urinalysis was positive for blood, but no red blood cells were present - myoglobin is often detected as blood on urine dipstick testing. Initial Clinical Institute Withdrawal Assessment (CIWA) scores ranged from 9 to 12, and he was treated per hospital protocol with lorazepam. Chest X-ray revealed patchy right lower lobe infiltrates (Figure 1).

**Figure 2:**
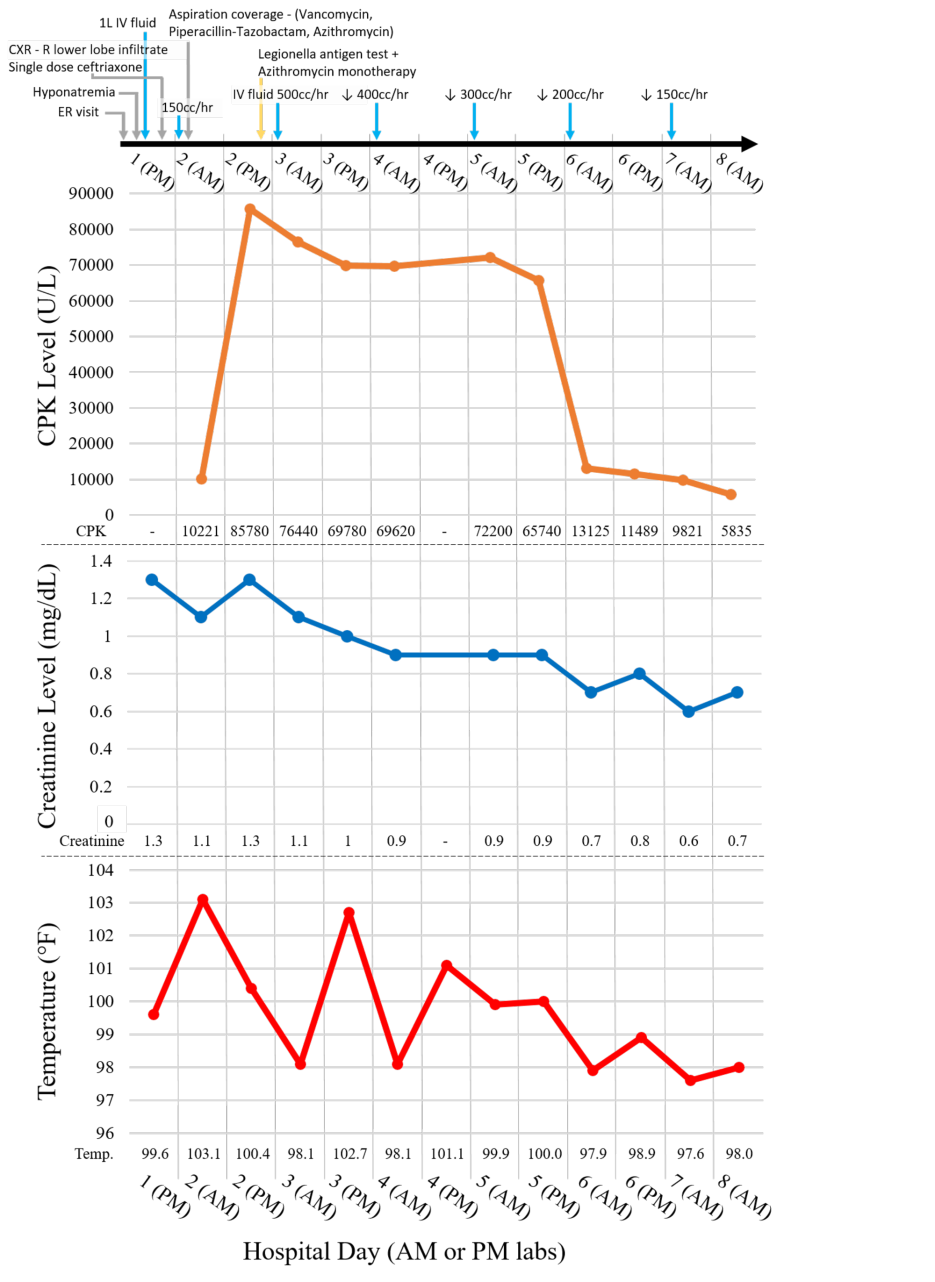
Treatment Course and Selected Lab Trends Graphs showing the time course of the patient’s treatment with interventions and selected labs. Time course displayed as follows: hospital day # (AM vs. PM lab draw). Azithromycin and IV fluids were stopped prior to the patient’s discharge home on hospital day nine. CPK: creatinine phosphokinase; ER: emergency room.

The patient was treated with IV hydration, thiamine, folic acid, vitamin K, and IV ceftriaxone for community-acquired pneumonia. On hospital day two, he developed worsening leukocytosis to 14.82x10^3 cells/µL (3.6-11) and fever to 103.1 °F, and antibiotics were broadened to vancomycin, piperacillin-tazobactam, and azithromycin. The CPK rose to 10,221 U/L (0-250) on the morning of day two, peaked at 85,780 U/L late on day two, and remained elevated in the 65,000 to 76,000 U/L range through hospital day five; there was a concurrent rise in transaminases with a high AST/ALT ratio (AST:ALT 115:53 units/L on admission, trending upwards to peak of 451:213 units/L on hospital day six) (Figure 2).

He was treated for rhabdomyolysis with aggressive IV hydration. Creatinine peaked at 1.3 mg/dL on hospital day two, and normalized at 0.9 mg/dL by hospital day five. Early on hospital day three, the Legionella urinary antigen returned positive (sputum cultures were also ordered and eventually tested positive, polymerase chain reaction (PCR) was not ordered), and the patient was switched to azithromycin monotherapy. On day five he defervesced, and on day six the CPK dropped dramatically to 13,125 U/L, and continued to decline steadily. A similar pattern was seen with the transaminases. White blood cell (WBC) count trended consistently down from admission, maintaining the lowest range counts of 5.42-6.05 x10^3 cells/µL between hospital days four through six (leukocytosis resolved between hospital days two and three). Blood, urine, and stool cultures and a Clostridium difficile PCR were all negative; the diarrhea resolved, and his hemodynamic and respiratory status remained stable throughout the hospitalization. He was discharged on hospital day nine after completing a six-day course of azithromycin.

## Discussion

Legionella infection can cause an acute, self-limited febrile illness (Pontiac fever), or pneumonia (Legionnaires' disease). Our patient presented with classical findings associated with Legionnaires' disease, including GI symptoms (nausea, diarrhea), hyponatremia, transaminitis, pulse-temperature dissociation, and high fever, but without the classic neurological signs such as headache and confusion. The traditional patient population for Legionella includes the elderly, smokers and the immunocompromised [1]. Our patient was a heavy drinker, which has also been reported to be a risk factor for contracting Legionella pneumonia [2]. Legionella infection is a public health concern due to its spread via contaminated water sources. On review of potential infectious sources, the patient reported working odd jobs as a handyman with exposure to wet carpets. No other exposures or sick contacts were noted.

Rhabdomyolysis can be caused by a variety of factors, including muscular trauma or compression (crush injury, prolonged immobilization after falls), drugs, toxins, infections (including Legionella), and electrolyte disorders [3]. Laboratory diagnosis of rhabdomyolysis typically includes urinalysis with dipstick positivity for blood in the absence of red blood cells, elevated urine myoglobin levels, and CPK levels exceeding 20,000-30,000 U/L. The CPK usually rises within two to twelve hours of muscle injury, reaching maximum values at 24-72 hours [3]. In assessing the underlying cause of his rhabdomyolysis, our patient had two possible causes, both of which probably contributed. The timing of the CPK peak at 24 hours after the fall suggests that muscle injury from the fall and subsequent time down on the floor might have been the major cause of his rhabdomyolysis. However, the patient’s description of the event (less than one hour down, no focal pain) makes a massive muscular injury seem less likely. Additionally, CPK from muscle injury should fall at a relatively constant rate (40%-50% of the previous day’s value) [3]. Our patient’s CPK remained elevated at a fairly constant level for five days, raising suspicion for ongoing muscle injury, more likely from the Legionella infection. Also, the patient’s cyclic fevers had largely resolved by hospital days five to six, corresponding well with the dramatic drop in CPK, which also supports an infectious cause. Legionella has been reported to cause rhabdomyolysis [4-5], severe enough to cause renal damage and even death. While the mechanism of Legionella-associated rhabdomyolysis is unknown, it is thought to be either release of an endotoxin in the systemic circulation with subsequent muscle and kidney injury, or direct invasion of legionella into the muscle tissues [6]. Taken together, the lack of evidence of severe muscle trauma in the initial episode, the rapidly peaking CPK that correlated with the patient’s highest fever, the sustained CPK elevation over five days, and the rapid drop in CPK that coincided with clinical signs of resolution of the infection all suggest that Legionella infection was the primary cause of the rhabdomyolysis.

There are a number of case reports of Legionella-associated rhabdomyolysis causing acute kidney injury, in most cases requiring dialysis. A PubMed MeSH search with the terms “Legionella”, “rhabdomyolysis”, “renal failure” and “kidney injury”, yielded 15 cases in addition to ours (Table 1).

**Table 1:** Comparison of Cases in Literature Reporting Legionella with Rhabdomyolysis Table comparing current case to those found in the literature review, focusing on requirements for dialysis, creatinine kinase & creatinine peaks and end result of clinical situation. Key: * = only value found in paper, may not reflect true peak. AKI: acute kidney injury; HIV: human immunodeficiency virus; COPD: chronic obstructive pulmonary disease; ATN: acute tubular necrosis; ARDS: acute respiratory distress syndrome.

Study	Age	Dialysis Required	CK Peak (U/L)	Creatinine Peak (mg/dL)	End Point	Notes
Buzzard et al. (this case)	53	No	85,780	1.3	Recovery	Rhabdomyolysis without significant AKI
Agu et al. (poster abstract)	45	No	159,466	Elevated, Unknown	Recovery	Rhabdomyolysis, AKI
Labidi et al. [7]	39	No	3,276	11.05	Recovery	Rhabdomyolysis, AKI w/ rapid recovery (fluids, antibiotics)
McConkey et al. [6]	56	No	Elevated, Unknown	Elevated, Unknown	Recovery	Rhabdomyolysis, AKI, Presenting CK 5141, Creatinine 1.9
Hall et al. [4]	26	Yes	165,600	17.7	Recovery	Rhabdomyolysis, Myoglobinuria, AKI, Sickle Cell Trait
Seegobin et al. [5]	51	Yes	51,092	13.04	Recovery	Rhabdomyolysis, AKI, HIV, COPD
Koufakis et al. [8]	45	Yes	82,026	10	Recovery	Rhabdomyolysis, AKI, Past Splenectomy
Shimura et al. [9]	54	Yes	52,656	5.1	Recovery	Rhabdomyolysis, ATN, AKI
Shah et al. [10]	26	Yes	Elevated, Unknown	Elevated, Unknown	Recovery	Rhabdomyolysis, AKI
Erdogan et al. [11]	67	Yes	Elevated, Unknown	Elevated, Unknown	Recovery	Rhabdomyolysis, AKI
Linga and Deo (abstract)	40	Yes	>20,000	Elevated, Unknown	Recovery	Rhabdomyolysis, AKI
Li et al. [12]	55	Yes	8,652	2.86	Recovery	Rhabdomyolysis, AKI, Acute Liver Failure
Narita et al. [13]	48	Yes	Elevated, Unknown	Elevated, Unknown	Recovery	Rhabdomyolysis, AKI, ARDS
Sposato et al. [14]	61	Yes	16,738	2.1*	Died	Rhabdomyolysis, AKI, Respiratory Failure
Matsumoto et al. [15]	67	Yes	5,068	3.8	Recovery	Rhabdomyolysis, AKI, Liver Dysfunction, Encephalopathy
Tokuda et al. [16]	57	Undetermined	70,455	8.2	Died	Rhabdomyolysis, ARDS, Septic Shock, likely AKI

Each of these Legionella cases indicated rhabdomyolysis as a major complication. Of the cases describing rhabdomyolysis, 11 required dialysis, three did not, and one patient died prior to initiation of dialysis [4-16] (Poster: Agu C, Basunia M, Salhan D et al. Legionella pneumonia associated with severe rhabdomyolysis and acute kidney injury. American Journal of Respiratory and Critical Care Medicine; May 18, 2016), (Poster: Linga K, Deo D. Pneumonia. Rhabdomyolysis and acute renal failure - a deadly cocktail! American Journal of Respiratory and Critical Care Medicine; May 18, 2014). Although CPK levels above 50,000 U/L were generally associated with higher peak creatinine levels, the height of the CPK peak did not necessarily correlate with the severity of the renal injury. Our patient, for example, had a relatively mild acute kidney injury (AKI) with peak creatinine 1.3 mg/dL despite a CPK of 85,000 U/L. As with the other four patients with Legionella pneumonia and rhabdomyolysis who did not require dialysis, our patient’s recovery might reflect normal renal function at baseline, aggressive early treatment with fluid resuscitation and antibiotics, or other unknown renal protective factors. A risk prediction model developed by McMahon et al. for AKI in all-cause rhabdomyolysis associates CPK >40,000 U/L with increased composite risk of severe AKI or death [17-18]. Additional predictors in this model include age, and initial creatinine, calcium, phosphorus, and bicarbonate levels. Our patient’s score based on this model was 8.5, which correlates with a relatively high risk for severe AKI or death [17-18]. The mainstay of treatment for prevention of AKI in rhabdomyolysis is aggressive isotonic fluid resuscitation with target urine output of 200-300 mL/hour, to be continued until the CPK level drops below 5000 U/L.

## Conclusions

Legionella pneumonia is a systemic infection that can be complicated by severe rhabdomyolysis and renal failure. The prognosis is generally worse in patients with very high CPK levels, but prompt treatment with appropriate antibiotics and aggressive hydration can lead to full recovery without dialysis in some cases. Legionella infection should be considered in acutely ill patients with rhabdomyolysis of unclear etiology.
